# Supplementary material for: Development of chemokine network inhibitors using combinatorial saturation mutagenesis
Source: Commun Biol. 2025 Apr 3;8:549. doi: 10.1038/s42003-025-07778-6 (PMC11969024; doi:10.1038/s42003-025-07778-6)
Supplement: Supplementary file 4 — Supplementary Data 2 [file 42003_2025_7778_MOESM4_ESM.zip › SupplementaryData3/scripts/xattune_dr.tar!/./script/analysis/JK/JK_E174_CCL19_ATC_plot.pdf]

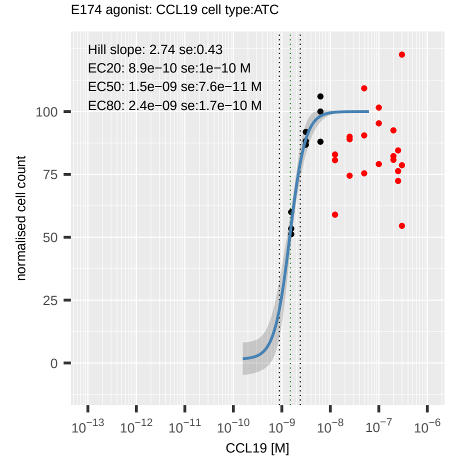

E174 agonist: CCL19 cell type:ATC
Hill slope: 2.74 se:0.43
EC20: 8.9e−10 se:1e−10 M
EC50: 1.5e−09 se:7.6e−11 M
EC80: 2.4e−09 se:1.7e−10 M
normalised cell count
100
75
50
25
0
10−13
10−12
10−11
10−10
10−9
10−8
10−7
10−6
CCL19 [M]
